# Production of hyperimmune serum against genotype VII Newcastle disease virus in rabbits with several applications

**DOI:** 10.5455/javar.2022.i586

**Published:** 2022-06-26

**Authors:** Dwi Desmiyeni Putri, Okti Nadia Poetri, Agung Adi Candra, Retno Damajanti Soejoedono

**Affiliations:** 1 Department of Animal Husbandry, Politeknik Negeri Lampung, Lampung, Indonesia; 2 Department of Clinic Reproduction and Pathology, Faculty of Veterinary Medicine, IPB University, West Java, Indonesia

**Keywords:** HI titer, hyperimmune serum, Newcastle disease

## Abstract

**Objective:**

This study aimed to produce hyperimmune serum against genotype VII Newcastle disease virus (NDV) with several applications.

**Materials and Methods:**

Production of hyperimmune serum against genotype VII NDV was performed on eight New Zealand white rabbits divided into four groups. Rabbits were immunized three times on the 1st day, the 14th day, and the 30th day. Blood sampling was carried out on the 8th day after the third immunization.

**Results:**

All groups showed the same pattern of hemagglutination inhibition (HI) titer results. HI titers would peak on the 5th or the 9th day after the second immunization, then decrease until the 3rd day after the third immunization, and increase again on the 5th day after the third immunization. Rabbits immunized intravenously showed higher HI titers than the other groups. These results indicate that the intravenous route for hyperimmune serum production against genotype VII Newcastle disease virus greatly affects the immune response result.

**Conclusions:**

The production of hyperimmune serum by intravenous immunization three times was able to produce the highest titer of 2^10^ at 38 days. The agar gel precipitation test and the Western blot assay showed that the hyperimmune serum was specific for the Newcastle disease antigen.

## Introduction

Avian paramyxovirus serotype-1 (APMV-1) which belongs to the paramyxoviridae family is the virus that causes Newcastle disease (ND) [1,2]. The Newcastle disease virus (NDV) can infect more than 250 species of birds, and infection by virulent strains can cause high morbidity and mortality with significant symptoms [3]. The wide range of susceptible hosts causes the persistence of NDV, which is endemic in many countries in the world. Virulent strains of infection have resulted in four panzootics [4]. The first outbreak of ND by a virulent strain happened in Java, Indonesia, in 1926, and at the same time, an outbreak occurred in England, precisely in the Newcastle upon Tyne region [5].

The Newcastle disease virus has a 15.2 kb enveloped, unsegmented, single-stranded RNA genome [5,6]. The NDV genome encodes six polypeptides, namely the nucleocapsid protein (NP), phosphoprotein (P) protein, matrix (M) protein, fusion (F) protein, hemagglutinin–neuraminidase (HN) protein, and the RNA-dependent RNA polymerase (L) protein. The virus nucleocapsid core consists of NP bound to RNA [7].

The Newcastle disease virus may vary widely in the severity of the disease in birds [8]. Multiple factors can contribute to the severity of disease, including species of host, immune status, age, environmental conditions, secondary infections, the number of viruses transmitted, the mechanism of transmission, and most importantly, the virulence of the infecting virus [9]. In comparison, susceptible species are chickens, whereas geese and ducks do not show symptoms; therefore, waterfowls act as the natural reservoir for NDV. The cleavage site of the F protein is a main determinant of viral virulence during replication in host cells. [10,11]. Based on the pathogenicity of the disease, ND can be classified into five pathotypes: neurotrophic velogenic strain, exhibiting respiratory and neurological symptoms with a high mortality rate; viscerotropic velogenic strain, causing hemorrhagic and highly pathogenic intestinal lesions; mesogenic strains caused by viruses with rare respiratory and neurological symptoms, while the age of susceptible birds is related to mortality; viral lentogenic strains present with mild respiratory infection; and asymptomatic enteric strain, exhibiting no clinical signs or asymptomatic [12].

Interaction between viruses and the environment, including the host immune system, resulted in NDV evolution and continues to produce new genotypes of viruses. Lately, infection of genotype VII NDV has caused high mortality of birds in several poultry farms in Indonesia [13,14]. Recently, producing hyperimmune serum in animals has become an essential activity of many research projects. The hyperimmune serum as a biological reagent will continue to be developed for research needs and possibly also for commercial applications in the future, such as for the therapy and the development of immunodiagnostic tools [15]. The specific antibodies in hyperimmune serum can be used to treat and control diseases in the event of an outbreak [16]. The hyperimmune serum is already used to successfully treat some diseases like foot and mouth diseases, tetanus, and canine viral diseases [17]. Currently, the hyperimmune serum used for diagnostics in poultry is imported from different countries of the world at very high prices. Moreover, the indigenous isolates may differ from the imported strains of viruses, showing nonspecificity in diagnosis [17]. The development of the serum for NDV currently circulating must be followed by the development of immunodiagnostic tests to obtain accurate test results. Therefore, it is necessary to produce genotype VII Newcastle disease hyperimmune serum that can be used as an immunodiagnostic reagent.

Hyperimmune serum production can be carried out in various applications, with or without adjuvants, with its own advantages and disadvantages. Considering the numerous applications of hyperimmune serum in research and clinical fields, the preparation method for developing hyperimmune serum against pathogens is essential [15]. To be able to produce antibodies with high titers in a short time, it is necessary to conduct research on various immunization applications with or without adjuvant-inducing immunity. This study aimed to produce hyperimmune serum against genotype VII NDV with several applications efficient in time and cost.

## Materials and Methods

### Ethical approval

This research has been approved by the Animal Care and Use Committee of IPB University’s Research and Community Services Institution. The approval number for this research is 213-2021 IPB.

### Newcastle disease antigen

For the production of NDV hyperimmune serum, characterized genotype VII NDV was used. The isolate was the repository sample from the Immunology Laboratory, Faculty of Veterinary Medicine, IPB University. We classified the isolate as genotype VII NDV based on PCR, sequencing, and phylogenetic analysis [11,13]. The antigen was prepared in fresh condition with and without adjuvant use.

### Hyperimmune serum production

This study used eight New Zealand white rabbits aged 2.5–3.5 months with an average body weight of 2,5 kg to produce hyperimmune serum against genotype VII NDV. We divided the rabbits into four groups. The first group was immunized subcutaneously with 1 ml of genotype VII NDV isolate (5 × 106^.5 ^ELD50/ml) and 1 ml of incomplete Freund’s adjuvant (IFA); the second group was immunized subcutaneously with 0.5 ml of genotype VII NDV isolate (5 × 106^.5 ^ELD50/ml) and 0.5 ml IFA; the third group was immunized with 1 ml of genotype VII NDV isolate (5 × 106^.5 ^ELD50/ml) subcutaneously; and the last group was rabbits immunized with 1 ml of genotype VII NDV isolate (5 × 106^.5 ^ELD50/ml) intravenously. Table 1 shows how the antigens used in this study.

We immunized rabbits three times. The first immunization was on the 1st day, the second immunization on the 14th day, and the third immunization on the 30th day. Blood sampling was carried out on the 8th day after the third immunization. The hyperimmune serum was collected by giving a local anesthetic agent into the ear and then taking blood from the intra-arterial arteries. The procedure for making serum is as follows: we kept blood samples at a temperature ±25°C for an hour and then kept them overnight at 4°C. The serum was separated manually and precipitated by centrifugation at 2,500 rpm for 15 min. Furthermore, we kept the serum in a collecting tube of 1.5 ml and stored it at -20°C until use. The rabbit blood samples were taken periodically to observe the hemagglutination inhibition (HI) antibody titer against genotype VII NDV. Serum was inactivated at 54°C for 30 min before being used for the HI test.

**Table 1.** Composition and application of genotype VII NDV isolate.

Group	Volume		Application
	Antigen	IFA^a^	
1	1 ml	1 ml	Subcutaneously
2	0.5 ml	0.5 ml	Subcutaneously
3	1 ml	-	Subcutaneously
4	1 ml	-	Intravenously

a Incomplete Fruend’s adjuvant.

### Serum purification

Purification of the hyperimmune ND serum was carried out in two stages. The first was precipitation by ammonium sulfate (4.1 M) [18]. The first stage of serum precipitation was to stir equal volumes of serum solution and ammonium sulfate slowly, then incubate them overnight at 4°C. After that, we centrifuged the precipitates at 3,000× g for 20 min. To obtain one-fourth of the antibody volume, we reconstituted the pellet with phosphate-buffered saline pH 7.4. Hereafter, dialysis was performed by putting the precipitate in a dialysis bag and stirring it in PBS pH 7.4 for 24 h at 4°C, which was replaced every 8 h by PBS solution. The second step was hyperimmune serum purification using a protein A purification kit (BioVision, USA) according to the manufacturer’s instructions.

### Serum characterization by SDS-PAGE

We measured the molecular weight of the purified ND hyperimmune serum using sodium dodecyl sulfate-polyacrylamide gel electrophoresis (SDS-PAGE). In SDS-PAGE, the concentration of separating gel was 12% and 4% for the stacking gel [19]. 5 µl of the sample buffer (containing bromophenol blue, SDS, DTT, and glycerol) was mixed with the serum sample (5 µl) and heated at 65°C for 5 min to denature the protein. 5 µl of marker protein (Thermo Scientific, USA) and 10 µl of hyperimmune serum samples were used. Protein separation was carried out using electrophoresis at 100 V for 150 min. The final process of serum characterization was staining the electrophoresis gel with Coomassie Brilliant Blue for 30 min and then adding a de-staining solution for 24 h.

### Serum confirmation by agar gel precipitation test and Western blot assay

The specificity of ND hyperimmune serum can be determined in several ways, including agar gel precipitation test (AGPT) and Western blot assay. ND antibody specificity was confirmed for two ND viruses [13] and other antigens, such as infectious bursal disease (IBD) and avian influenza (AI). The precipitation line in the agarose gel indicated antigen and antibody interaction.

To detect Newcastle disease viral protein, we ran the genotype VII NDV antigen on an SDS-PAGE gel. The SDS-PAGE result was transferred to nitro cellulose (NC) membranes. The membrane was blocked with Tris-buffered saline (TBS) at 37°C for 2 h. After the T-TBS washing, we incubated the membrane with a 1:2,000 dilution of primary rabbit hyperimmune serum (against NDV produced in this research) overnight, and then washed it with T-TBS. Afterward, we incubated the NC membrane in alkaline phosphatase-conjugated with a secondary antibody at 37°C for 2 h. Then, we washed and developed the membranes using a diaminobenzidine substrate solution (Sigma) for 5–10 min. At the end of this procedure, we washed the membrane with distilled water to terminate the enzyme reaction on the membrane.

## Results and Discussion

### Antigen preparation

The antigen used in this research was genotype VII NDV, characterized by PCR sequencing and phylogenetic analysis [11,13]. The ELD_50_ of a virus must be calculated to determine the virus’ ability to kill 50% of specific pathogen-free embryos in eggs. The virus used in this study is genotype VII NDV with 5 × 10^6.25^/ml ELD_50_. Before use, the virus must be filtered using a 0.45 µm filter. Antigen preparation was different depending on group treatment. The antigen was mixed with IFA before being used for the first and second groups. The antigen composition used was antigen: IFA in a 1:1 ratio. Shake the solution in a glass syringe with a connector to make an antigen–IFA emulsion.

### Production of hyperimmune serum against genotype VII Newcastle disease virus

The main purpose of hyperimmune serum production is to gain a high titer with high antibody specificity, which continues to cause concern in animal welfare. Hyperimmune serum production needs several animals as subjects to invasive treatments such as antigen injection and serum collection [20,21]. This study used rabbits as a donor for hyperimmune serum, which received invasive treatment, immunization, and serum collection. Because of its low cost–benefit ratio and ease of handling, rabbits are widely used as donor antibodies [22]. Moreover, rabbits are not closely related to chickens as a natural host of the Newcastle disease virus [20]. This study used eight female rabbits aged 2.5–3.0 months as biological agents for producing hyperimmune serum against genotype VII NDV. Hyperimmune serum production against genotype VII NDV was performed with and without adjuvants and applied subcutaneously and intravenously. Adjuvants work to increase the immune response through a “depot” effect mechanism that increases antigen presentation slowly. The adjuvant immunostimulant properties can harm the animals because they induce inflammation and tissue destruction, which potentially causes pain and distress [23]. The adjuvants used in this study was incomplete Fruend’s adjuvant (IFA) because it minimizes pain and distress in rabbits while retaining the potency as an immunostimulant agent.

**Figure 1.**
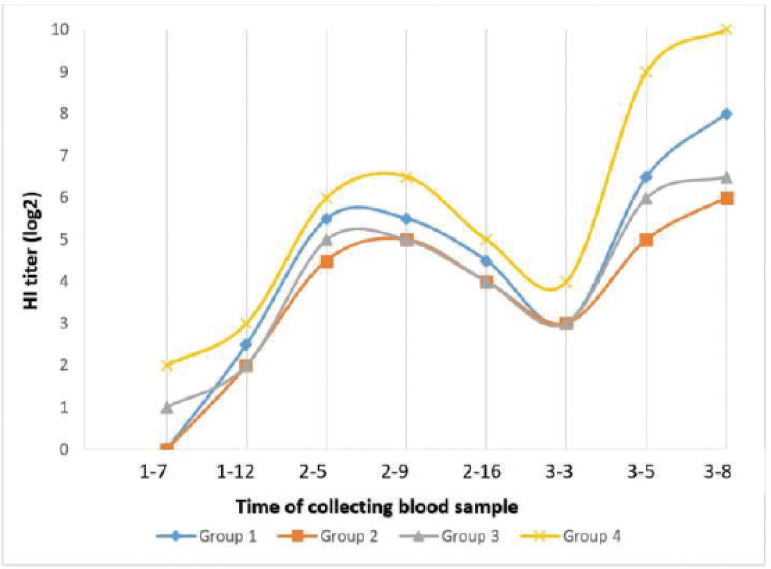
Hemagglutination inhibition titer after immunization.

Some factors can influence the efficacy of immunization. They are divided into three categories: (1) antigen, including formulation, adjuvant, and dose; (2) recipients of the vaccine; and (3) the route of immunization [24]. Hyperimmune serum against genotype VII NDV was produced in several applications. For hyperimmune serum production, we immunized the rabbits with antigen–IFA emulsion in the first and second groups, while the third and fourth groups did not use IFA in antigen preparation. Immunization in the first, second, and third groups was administered subcutaneously, while in the fourth group, immunization was administered intravenously. In the second group, antigen volume was half of the first group. The Newcastle disease hyperimmune serum generated in this study resulted from three immunizations to induce a higher HI titer. The first immunization is intended to introduce antigen into the immune system, particularly the B cell, whereas the second and third immunizations are boosters designed to modulate antibody production by B cells [25,26]. The second immunization was carried out on the 14th day after the first immunization, and the third immunization was on the 16th day after the second immunization. The hyperimmune serum titer against genotype VII NDV was measured with a periodic HI test, and hyperimmune serum was collected on the 8th day after the third immunization. The hyperimmune serum titer result is shown in Figure 1.

Based on Figure 1, the first group immunized with NDV–IFA emulsion showed that the HI titer was already detected on the 12th day after the first immunization and reached 2^5.5 ^on the 5th day and the 9th day and then decreased on the 16th day after the second immunization. The HI titer in this group continued to decline until the 3rd day after the 3rd immunization, then increased until the 8th day after the 3rd immunization, and reached a HI titer of 28. The second group receiving NDV–IFA emulsion (each volume 0.5 ml) showed hyperimmune serum against NDV genotype VII detected on the 12th day after the first injection, and the HI titer reached 2^4.5 ^on the 5th day after the second immunization and reached a peak on the 9th day after and then decreased on the 16th day. The HI titer in this second group continued to decline until the 5th day after the third immunization and then increased, reaching 2^6^ HI titer on the 8th day after the third immunization. The first and second groups were different in the dose of antigen and adjuvant, and these conditions influenced the HI titer result. The first and second groups have a difference in HI titer of about 2 log levels. The amount of antigen can change the immune response and, in turn, the number of antibodies made [20,21].

The group of rabbits that received NDV subcutaneously showed that an HI titer could be detected on the 7th day after the first immunization. This group showed the same HI titer as the second group until the 3rd day after the third immunization, except on the 5th day after the second immunization, when this group reached a HI titer of 25, which was higher than the second group. Furthermore, on the 5th day after the third immunization, this group showed an antibody titer reaching 2^6^, and at the end of the serum collection on the 8th day after the third immunization, the HI titer reached 2^6.5^. The group of rabbits that received genotype VII NDV subcutaneously showed the same HI titer pattern as the second group, but with higher HI titers. The second and third groups differ in antigen volume and adjuvant. Immunization using a half dose (volume) and mixture with adjuvants produced almost the same antibody titer as immunization using a full dose of antigen only (without adjuvant). The difference occurs at the beginning of antibody formation. The antibody formation process in the group that received NDV–IFA emulsion needed more time. Furthermore, at the end of the hyperimmune serum production, the group that received NDV–IFA emulsion showed a 1 log higher HI titer. For the secondary and booster injections, we used incomplete Freund’s adjuvant as a water-in-oil emulsion with antigen to raise polyclonal and monoclonal antibodies [23]. Awate et al. [27] stated that compared to injection of antigen alone, injection of antigen plus an adjuvant generally permits the use of a much smaller quantity of antigen, while greatly enhancing the serum antibody response. The adjuvants promote an increased immune response slowly [23,27]. In general, adjuvants permit the use of smaller antigens but still retain the ability to modulate the immune response against the antigen. Samiullah et al. [28] produced an antibody for APMV-1 using adjuvant within 91 days and reach a 1024 (2^10^) HI titer with 4 and 5 injections. Putri et al. [29] produced antibodies to Newcastle disease in New Zealand rabbits with subcutaneous route application for the first and second injection, which resulted in the same pattern of antibody titer until the 16th day after the second injection. Moreover, after the third injection intravenously, the study revealed a higher antibody titer on the 8th day, reaching HI titer of 2^9^.

The last group of rabbits, immunized by antigen Newcastle disease intravenously, showed that HI titer started to be detected on the 7th day after the first immunization and reached HI titer 2^6^ on the 5th day after the second immunization. It continued to increase until the 9th day, with the HI titer reaching 2^6.5 ^and decreasing on the 16th day. The HI titer continued to decrease until the 5th day after the third immunization and then increased on the 8th day after the third immunization and achieved a 2^10 ^HI titer. Rabbits receiving intravenous immunization showed higher antibody titers than the other groups. These results indicate that the intravenous route application for hyperimmune serum production against the genotype VII NDV dramatically affects the immune response result. The intravenous route has the potential for a broad distribution of antigens. Intravenous routes will distribute the antigen, firstly to the spleen and secondarily to the lymph nodes. Intravenous may be the most effective route for small particulate antigens, such as cells, virions, or bacteria [30].

### Serum purification

The serum is a blood component that contains albumin and globulin proteins [31]. The serum component that can bind directly to the antigen is called an antibody [32]. Before being characterized, the serum must be purified from other components. Some purification methods could be used to separate serum [33]. In this study, we purified the hyperimmune serum with ammonium sulfate (4.1 M) and a protein A purification kit (BioVision). Ammonium sulfate is the oldest, easiest, and most economical method used most frequently to precipitate and thus concentrate immunoglobulins from the serum [34]. The principle of ammonium sulfate purification is the ability of ammonium sulfate to bind immunoglobulin G (IgG) [35]. The second stage of hyperimmune serum purification was using a protein A purification kit. Protein A, located in the surface protein of *Staphylococcus aureus* [36], has five domains that have the ability to bind the Fc fragment of IgG [37]. After the protein purification, it is important to know the protein concentration in our samples. In this study, we determined the antibody concentration in serum by a UV-Vis spectrophotometer at a 280 nm wavelength. Based on the UV-Vis spectrophotometer result, the genotype VII ND antibody concentration is 1.97 μg/μl.

### Serum characterization by SDS-PAGE

**Figure 2.**
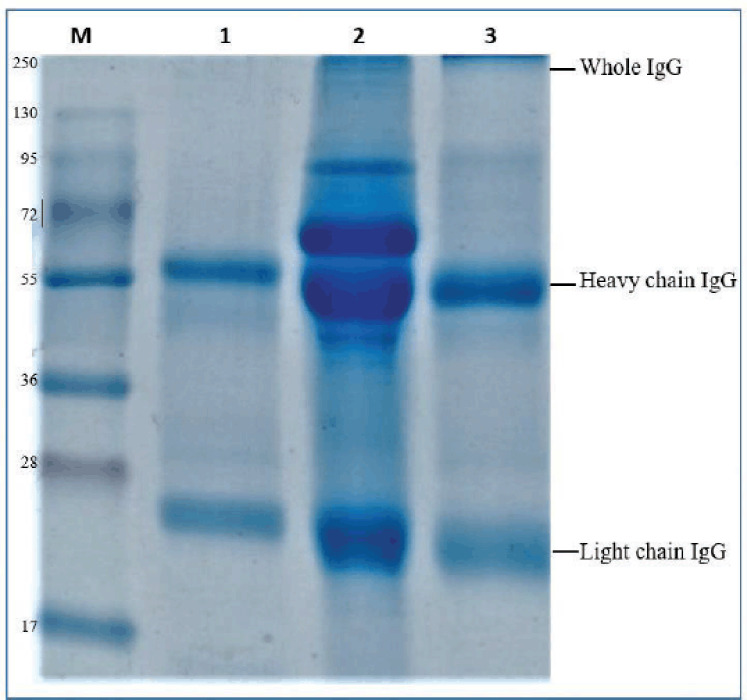
SDS-PAGE result of ND hyperimmune serum. (M) Protein marker; (1) commercial standard antibody; (2) after purification by ammonium sulfate; and (3) after purification by the protein purification kit A.

SDS-PAGE was used to determine the protein profile and the molecular weight of hyperimmune serum against the genotype VII NDV. The SDS-PAGE result showed that purified serum by ammonium sulfate contained five protein bands, and the serum that had passed through two stages of purification only contained two protein bands, the same as with a standard commercial antibody (Fig. 2).

We determine the molecular weight of serum protein on SDS-PAGE by forming a linear curve based on calculating the relative mobility value (Rf) and the logarithm of the molecular protein weight. Based on the data in Table 2, a linear regression curve with equation *y* = -0.1134*x* + 2.2379 and R² = 0.9429 was obtained. The equations were used to determine the molecular weight of the standard antibody and purified serum samples, which are presented in Table 3.

**Table 2.** The migration distance from the marker along with the Rf value.

Rf (cm)	MW (kDa)	Log MW
0.14	250	2.40
0.96	135	2.13
1.71	95	1.98
2.65	72	1.86
3.76	55	1.74
5.52	36	1.56
6.9	28	1.45
9.69	17	1.23

*y* = -0.1134*x* + 2.2379; *R*² = 0.9429.

**Table 3.** The migration distance and molecular weight of the hyperimmune serum against Newcastle disease virus.

Rf (cm)	Log MW	MW (kDa)
Purification by ammonium sulfate		
0.43	2.19	154.57
2.12	2.00	99.42
3.95	1.79	61.66
4.44	1.73	54.25
7.51	1.39	24.34
Purification by Protein A		
4.44	1.73	54.25
7.51	1.39	24.34

Based on the regression equation calculation, we found that the molecular weight of the antibody standard was 154.57 kDa for whole IgG; the heavy chain was IgG 54.25 kDa; and the light chain IgG was 24.34 kDa. Immunoglobulin G had a molecular weight of 150–160 kDa [36].Chemical treatments such as SDS will break the IgG molecule by the disulfide bond, causing the polypeptide to break into four separate chains. These chains are “heavy” chains with a molecular weight of 50 kDa and “light” chains with a molecular weight of about 25 kDa. In the serum purified by ammonium sulfate only, we detected two banned proteins that were not the same as the standard antibody in molecular weight: 99.42 kDa and 61.66 kDa. Albumin is a protein found in serum with a molecular weight of 60 kDa [37]. In serum that has passed through two purification stages, it only has two protein bands that are the same as standard antibodies.

### Serum confirmation by agar gel precipitation test and Western blot assay

**Figure 3.**
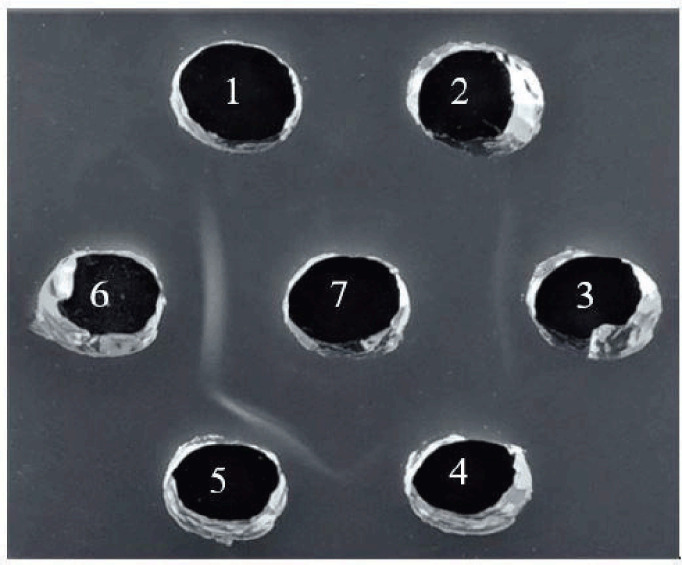
Serum confirmation with the agar gel precipitation test. (1) Avian influenza (Ag); (2) IBD (Ag); (3) Newcastle disease virus (Lasota); (4) Newcastle disease virus (Sato); (5) Newcastle disease virus genotype VII (1); (6) Newcastle disease virus genotype VII (2); and (7) hyperimmune serum. Arrow: precipitation line.

Serum confirmation is carried out to ensure that the antibodies contained in the hyperimmune serum against NDV are only able to bind to NDV. Several methods can confirm this, including AGPT and Western blot assay. The agar gel precipitation test has been applied to detect precipitating antibodies in various avian diseases. The confirmation results of the ND antibody specificity can be seen in Figure 3.

The antigen–antibody interaction on AGPT was characterized by a precipitation line in the agarose gel. The agar gel precipitation test result showed the line of precipitation formed on all ND antigens, whereas in wells given the avian influenza and the IBD antigen, we could not find the precipitation line. This result indicated that this research’s hyperimmune serum against Newcastle disease virus has specificity.

**Figure 4.**
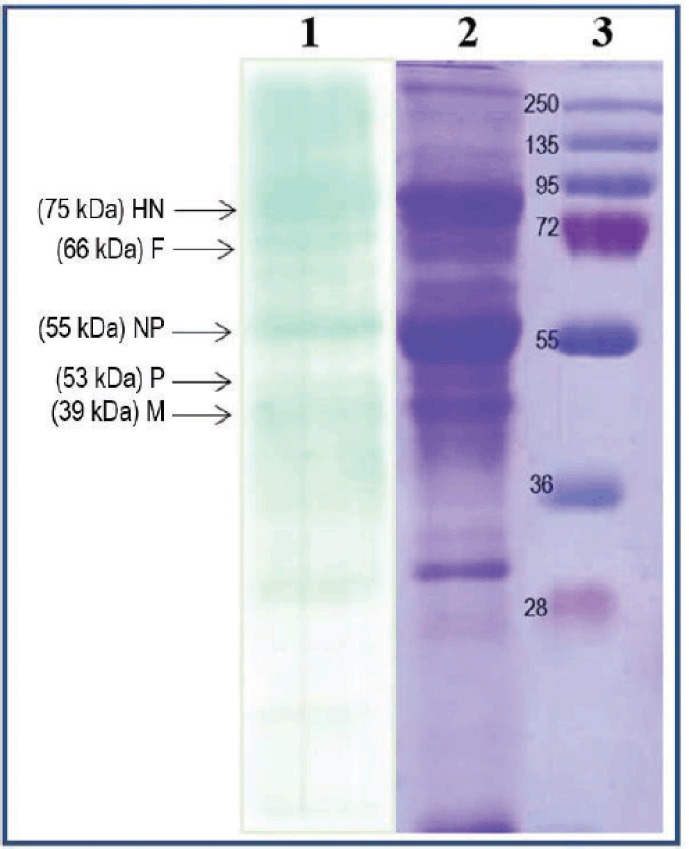
Western blot assay antigen–antibody Newcastle disease. (1) Western blot assay result; (2) SDS-PAGE result of Newcastle disease virus; and (3) protein marker.

In addition to AGPT, the Western blot assay was also used to confirm whether the antibody in the Newcastle disease serum produced could bind to Newcastle disease virus proteins. By using the Western blot method, researchers can identify specific proteins from a complex mixture of proteins extracted from cells [40]. This stage begins with the separation of viral proteins with SDS-PAGE, followed by the transfer of viral proteins to nitrocellulose membranes. The Western blot assay results are shown in Figure 4.

Based on the SDS-PAGE results of NDV, 5–8 proteins were recorded with a molecular weight ranging from 28 to 200 kDa. To know the molecular weight of each protein band, the relative mobility must be determined first and then entered into the following equation: *y* = -0.1134*x* + 2.2379; R² = 0.9429. Table 4 shows the molecular weight of the Newcastle disease protein, which we got from the regression equation.

Hemmatzadeh and Kazemimanesh [41] detected Newcastle disease proteins HN, F, NP, P, and M with molecular weights of approximately 75, 66, 55, 53, and 39 kDa, respectively, and that Western blot assay can detect those proteins. This shows that the antibodies made in this study could find the protein caused by the Newcastle disease virus.

**Table 4.** The molecular weight of Newcastle disease protein by SDS-PAGE.

RF (cm)	Log MW	MW (kDa)
3.19	1.876154	75.19
3.67	1.821722	66.33
4.36	1.743476	55.40
4.52	1.725332	53.13
5.67	1.594922	39.35

The main goal of antibody production is to obtain high-titer, high-specificity antibodies while still being concerned about the animal welfare. The study successfully produced the hyperimmune serum for Newcastle disease in rabbits. Normal antibodies can be replaced with hyperimmune serum, which can also be used to test for viruses [17].

Hyperimmune serum can be used for large-scale screening of NDV-carrying commercial and wild birds [17]. The hyperimmune serum against NDV can be used to decrease the morbidity and mortality rate in experimentally infected birds [16]. Passive immunization against Newcastle disease has also been attempted with promising results. The symptoms of ND in experimentally infected birds with NDV are successfully treated through passive immunization using HIS [42]. The high doses of antibodies are also helpful in providing passive immunity by decreasing the mortality and morbidity in birds previously exposed to the ND virus of velogenic strain. With an increasing dose of HIS, mortality and morbidity are considerably reduced [42]. 

## Conclusion

The various application methods successfully produced hyperimmune serum against the genotype VII Newcastle disease virus. The production of hyperimmune serum by three intravenous immunizations produced the highest titer of 2^10^ at 38 days. The hyperimmune serum has specificity for Newcastle disease antigen based on the AGPT and Western blot assay results.

## List of abbreviations

NDV, Newcastle disease virus; HI, Hemagglutination inhibition; IFA, Incomplete Freund’s adjuvant; ELD, Embryo lethal dose; AGPT, Agar gel precipitation test; SDS-PAGE, sodium dodecyl sulfate-polyacrylamide gel electrophoresis.
